# First, do no harm: the proposed definition of “terminal anorexia” is fraught with danger for vulnerable individuals

**DOI:** 10.1186/s40337-022-00605-x

**Published:** 2022-06-16

**Authors:** Megan Riddle, Anne Marie O’Melia, Maryrose Bauschka

**Affiliations:** 1 Department of Psychiatry and Behavioral Sciences, University of Washington School of Medicine, Seattle, WA 98195 USA; 2 Department of Psychiatry, University of Utah School of Medicine, Salt Lake City, UT 84108 USA; 3 Department of Psychiatry, University of Utah School of Medicine, Salt Lake City, UT 84108 USA; 4 Department of Psychiatry, University of Colorado School of Medicine, 13001 E 17th Pl, Aurora, CO 80045 USA

**Keywords:** Anorexia nervosa, Terminal anorexia, Medical assistance in dying, Capacity, Physician-assisted suicide, Severe and enduring anorexia nervosa, Severe and enduring eating disorders

## Abstract

A recent article in the Journal of Eating Disorders (10:23, 2022) proposed criteria for “terminal anorexia” with a cited goal of improving access to end-of-life care (Gaudiani et al. in J Eat Disord 10(1):23, 2022). The authors presented three cases in which patients received end-of-life care, including the prescription of medical assistance in dying (MAID), also known as physician-assisted suicide (PAS). The proposed criteria lack the evidence base for adoption and do not acknowledge the compelling evidence that exists surrounding possible prolonged timelines to recovery for some individuals and the nuances of assessing capacity in this population.

## Main text

Primum non nocere. In an effort to avoid harm to the loved ones of the individuals involved, this commentary will not discuss specifics of the cases presented in Gaudiani and colleagues’ article, “Terminal anorexia: three cases and proposed clinical characteristics”. We appreciate the authors’ initiation of a discussion as to how to best support individuals who suffer profoundly from anorexia nervosa (AN) and its effects over a prolonged period of time, as this conversation is much needed in the field. However, the proposed clinical criteria for “terminal” anorexia nervosa are overly broad. These criteria are specified as (1) a diagnosis of anorexia nervosa, (2) age 30 or older, (3) prior engagement in high-quality and multidisciplinary eating disorder treatment and (4) capacity to choose death as an outcome of their illness. We highlight the risks posed to individuals with anorexia nervosa by labeling patients as “terminal” without a basis in evidence.

## Potential pitfalls of the first three proposed criteria

The field has long called for a unified definition of severe and enduring anorexia nervosa (SEAN) and a variety of markers related to chronicity and severity have been proposed [1, 2]. We wholeheartedly agree that the field needs to determine characteristics and risk factors that may predict lack of recovery in anorexia nervosa, however, we would argue that this article does not offer evidence-based criteria for what is described as “terminal anorexia.” While age over 30 has been proposed elsewhere as a marker of increased risk of the development of chronic illness [3], studies have suggested that recovery can occur, even well after two decades of struggling with an eating disorder [4]. In addition, attempts to stratify risk of long-term illness based on clinical characteristics have been of limited utility; while we may be able to predict at a population level who is at increased risk of developing SEAN, we lack the ability to predict whether a specific individual will develop enduring illness or be able to make steps towards recovery [5, 6]. Thus, labeling these patients as terminal robs them of the opportunity for treatment that could improve their quality of life. Also, while the vast majority of individuals who develop anorexia nervosa do so at ages younger than 30, there are cases that present later in life or persons who do not seek or who may be unable to access treatment until they are in their 30 s or later [7]. This then risks the label of “terminal” in the context of either a short duration of illness and/or simply not enough time spent in treatment to engender true brain changes towards recovery. From clinical experience, many patients require several treatment episodes spanning many years, or even decades, to gain traction in recovery [4]. Additionally, the various barriers that can arise to accessing high-quality care including cost and insurance barriers, limited or no local resources, and personal hardships that may be posed by taking time away from their daily life may change over time for an individual [8]. Recovery from anorexia nervosa can and does occur after the age of 30 and thus setting an arbitrary age criteria without an evidence base may limit patients' ability to receive the care they need.

## Criteria 4 and the challenge of capacity assessments in anorexia nervosa

Additionally, the article fails to fully acknowledge the challenges of assessing capacity specific to patients with anorexia nervosa. Patients with anorexia nervosa are often adept at listing back the risks, benefits and alternatives to the proposed treatment, and expressing their choice, but fail to appreciate how the information applies to them [3, 9]. Cognitions and behaviors secondary to anorexia nervosa are ego-syntonic, which adds to the challenge, and there is qualitative work to suggest that values regarding life and death differ between healthy individuals and those struggling with anorexia nervosa [10, 11]. There also is no acknowledgement of the impact of malnutrition on the brain [12], and how the eating disorder itself can usurp a patient’s autonomy [13], which would also be crucial to account for in a capacity evaluation. Rather, this article describes the use of local psychiatrists to assess capacity—who may or may not have expertise in this area [14, 15]—while negating the importance the role of ethics committees in the care and clinical decision-making of such complex patients. It will be crucial to identify reliable methods for determining capacity in this population, particularly with respect to end-of-life care decisions, through research and input from stakeholders including individuals with the lived experience of an eating disorder, their loved ones, and treating providers.

## The lack of objective data to define terminality

Central to medical assistance in dying (MAID), also known as physician-assisted suicide (PAS), is a terminal diagnosis. While receiving nutrition is immensely distressing to an individual with an eating disorder, it is critical to note that malnutrition is not a terminal illness and nearly all of the resulting medical complications can be reversed with nourishment [16]. The authors themselves note that, “there are no explicit physiologic markers or measurables (weight, degree of weight loss, presence of or degree of organ failure, vital signs) which delineate someone with terminal AN “ and that “the human body can be exceptionally resilient even with terminal malnutrition.” In the setting of no identified physiologic markers or measurables, or data, how can one predict whether or not an individual with anorexia nervosa has a less than six-month prognosis, and without this objective data is “terminal” the appropriate descriptor?

## The need to expand resources and expertise beyond higher levels of care

This paper does highlight the lack of resources and guidance available to patients with severe and enduring illness. While eating disorder treatment centers frequently have expertise in the management of these patients, ranging from a focus on recovery to shifting towards harm reduction and palliative care, individuals with anorexia nervosa may not have access to the same level of expertise in their community when they return home. We, as specialists in this complex and paradoxical illness, need to increase support for our colleagues in the long-term management of these patients.

## Conclusions

We again thank the authors of the article under comment for prompting a much-needed discussion about how to identify and best support individuals with anorexia nervosa who ultimately may be less likely to recover from their illness. It is true that some patients may find continued treatment more distressing than making the decision to die from their illness, and the eating disorder field needs to acknowledge and identify ways to address this lived experience. Patients with chronic psychiatric illness in general are a highly vulnerable population, and feelings of helplessness and discouragement are inherent in the battle for recovery; it is common for patients to repeatedly “fail” treatment before making progress towards recovery. Since this article was published, we have already had patients approach us to ask if we feel their case is “terminal.” If providers do not hold the hope for these patients when they can’t do so for themselves, use evidence to guide our recommendations, and acknowledge our own limits in knowing what the future holds for them, what will become of these vulnerable individuals?

## Data Availability

Not applicable.

## Abbreviations

SEAN: Severe and enduring anorexia nervosa
MAID: Medical assistance in dying
PAS: Physician-assisted suicide
